# Fluid–structure interaction modelling of a positive-displacement Total Artificial Heart

**DOI:** 10.1038/s41598-023-32141-2

**Published:** 2023-04-14

**Authors:** Joseph Bornoff, Azad Najar, Libera Fresiello, Thomas Finocchiaro, Ina Laura Perkins, Harinderjit Gill, Andrew N. Cookson, Katharine H. Fraser

**Affiliations:** 1 Department of Mechanical Engineering, University of Bath, Bath, UK; 2 Scandinavian Real Heart AB, Västerås, Sweden; 3 Faculty of Science and Technology, University of Twente, Twente, The Netherlands; 4 Centre for Therapeutic Innovation, University of Bath, Bath, UK

**Keywords:** Biomedical engineering, Mechanical engineering, Cardiac device therapy

## Abstract

For those suffering from end-stage biventricular heart failure, and where a heart transplantation is not a viable option, a Total Artificial Heart (TAH) can be used as a bridge to transplant device. The Realheart TAH is a four-chamber artificial heart that uses a positive-displacement pumping technique mimicking the native heart to produce pulsatile flow governed by a pair of bileaflet mechanical heart valves. The aim of this work was to create a method for simulating haemodynamics in positive-displacement blood pumps, using computational fluid dynamics with fluid–structure interaction to eliminate the need for pre-existing in vitro valve motion data, and then use it to investigate the performance of the Realheart TAH across a range of operating conditions. The device was simulated in Ansys Fluent for five cycles at pumping rates of 60, 80, 100 and 120 bpm and at stroke lengths of 19, 21, 23 and 25 mm. The moving components of the device were discretised using an overset meshing approach, a novel blended weak–strong coupling algorithm was used between fluid and structural solvers, and a custom variable time stepping scheme was used to maximise computational efficiency and accuracy. A two-element Windkessel model approximated a physiological pressure response at the outlet. The transient outflow volume flow rate and pressure results were compared against in vitro experiments using a hybrid cardiovascular simulator and showed good agreement, with maximum root mean square errors of 15% and 5% for the flow rates and pressures respectively. Ventricular washout was simulated and showed an increase as cardiac output increased, with a maximum value of 89% after four cycles at 120 bpm 25 mm. Shear stress distribution over time was also measured, showing that no more than $4.5\times 10^{-4}$% of the total volume exceeded 150 Pa at a cardiac output of 7 L/min. This study showed this model to be both accurate and robust across a wide range of operating points, and will enable fast and effective future studies to be undertaken on current and future generations of the Realheart TAH.

## Introduction

Heart failure (HF) affects more than 64 million people worldwide, with cases increasing by almost 92% between 1990 and 2017^1^. Severe cases of HF (New York Heart Association Class IV symptoms^2^), such as end-stage biventricular HF that affects both sides of the heart, require heart transplantation. However, the number of available donor hearts is limited and the transplant waiting lists continue to grow^3^. Doctors can turn to mechanical circulatory support (MCS) as a means to bridge the gap to transplant, and the type of HF dictates what MCS can be used. In cases of single ventricle failure, a Ventricular Assist Device (VAD) can be used to aid the ventricle to pump blood, however, in cases of end-stage biventricular HF, a Total Artificial Heart (TAH), which entirely replaces the function of the native heart, is more appropriate^4^.

There are two main pumping methods of MCS devices: rotary and positive-displacement. The most recent VADs are rotary devices^5–7^. These contain a single rotating impeller giving kinetic energy to the blood which is converted to pressure head by either stator blades (in an axial flow pump) or a volute (in a centrifugal flow pump). Rotary pumps usually produce a continuous flow, but by varying the impeller rotational speed they can be made to produce a pulsatile flow waveform. TAHs usually employ a positive-displacement pumping method, in which the blood is pushed from the device by a membrane or pusher plate, driven electrically or pneumatically, to produce pulsatile flow^8–10^. Rotary pumps have also been used as TAHs^11,12^ and positive-displacement pumps have also been used as VADs^13–16^. Whilst continuous flow devices are generally smaller than pulsatile pumps and have been shown to be more robust and reliable as compared with early positive-displacement VAD technology, studies have shown physiological benefits of pulsatility, both within the device and throughout the body^17,18^.

On the market there is currently only one TAH—the Syncardia TAH (Syncardia Systems, Tucson AZ, USA), approved by the FDA as a bridge to transplant device, it is a pneumatically driven positive-displacement TAH that produces pulsatile flow of more than 9 L/min^8^. However, it suffers from driveline infections and thromboembolic related events^19^. Several other devices are progressing towards approval and hope to overcome the issues with the Syncardia TAH. The CARMAT Aeson TAH (Carmat, Paris, France) is another positive-displacement pump that aims to improve biocompatibility by using a biological membrane and bioprosthetic heart valves^9^. It has recently been awarded the CE mark in Europe and has gained approval for early feasibility studies in the United States^20^. The ReinHeart TAH (ReinHeart, Aachen, Germany) uses a mechanical actuated pusher-plate to alternate pumping between two artificial ventricles, a different approach compared to the Syncardia (pneumatic) and CARMAT (hydraulic)^21^. The Cleveland Clinic CFTAH (Cleveland Clinic, Cleveland OH, USA) and the BiVACOR TAH (BiVACOR, Houston TX, USA) are both rotary TAHs that provide both systemic and pulmonary flow using a single moving rotor suspended by a hydrodynamic and maglev bearings respectively^11,12^. The Realheart TAH (Scandinavian Real Heart, Västerås, Sweden) is a novel four-chamber, two-sided pump that can deliver upwards of 7 L/min, and mimics the mechanics of the native heart by translating the atrioventricular (AV) plane to produce pulsatile flow, the direction of which is governed by a pair of bileaflet mechanical heart valves (BMHVs)^10,22^.

Computational fluid dynamics (CFD) is a vital tool that has been used to investigate the flow characteristics and performance of both VADs and TAHs when full experimental measurements are not possible, with rotary devices amongst the most commonly simulated^23–25^. The haemolytic potential of the Cleveland Clinic CFTAH was reduced through simulations of different right impeller designs^26^, using a previously validated CFD model that coupled electromagnetic and fluid flow solutions^27^. Some positive-displacement left ventricular assist devices (LVADs), including the Penn State LVAD^28^ have also been studied numerically using similar approaches to those used on TAHs^29–31^. Using pre-defined displacement of the membrane and monoleaflet valves, a computational model showed that smaller models of the Syncardia TAH were more likely to encounter elevated shear stress levels, owing to increased pumping frequencies and smaller stroke volumes^32^. For the CARMAT TAH, a fluid–structure interaction (FSI) simulation was first undertaken to obtain membrane and valve displacement, and was subsequently followed by a CFD simulation to investigate shear stress within the device^33^. This model was then used to investigate numerical washout of the device^34^, and damage to blood components^35^. Simulations of the ReinHeart TAH were also undertaken using a partitioned FSI approach to achieve a stable method^36^, and the method was later used to investigate the washout of the device with different orientations of the inlet valve^37^.

A CFD study of the Realheart TAH has been undertaken previously, where Kelly et al.^38^ used an immersed boundary FSI method to assess different approaches to capturing the BMHV motion within the device. They concluded that using in vitro data of the valve motion, captured using video analysis, returned suitable results at a given operating point. However, this approach was limited to simulating only the conditions where in vitro data were available. In addition, the immersed boundary method could not accurately resolve the valve leaflet–fluid interface, leading to poor shear stress resolution in this area. We previously developed a new valve motion modelling method that used an overset mesh, a novel blended weak–strong coupling method and variable time stepping to reliably capture the FSI motion of mechanical heart valves found within positive-displacement blood pumps^39^. This approach did not require pre-existing in vitro valve motion data for the model to work, and instead could be applied to a variety of operating conditions. The method was tested on a simple cylindrical pump in which the proximal valve translated and the distal valve remained in a fixed position.

The aim of this study was to employ this valve motion modelling method for a complete device model of the Realheart TAH. The variation in flow rate, pressure, valve kinematics, shear stress and fluid washout of the device was assessed for a variation in heart rate and stroke length.

## Methods

### Computational domain

**Figure 1:**
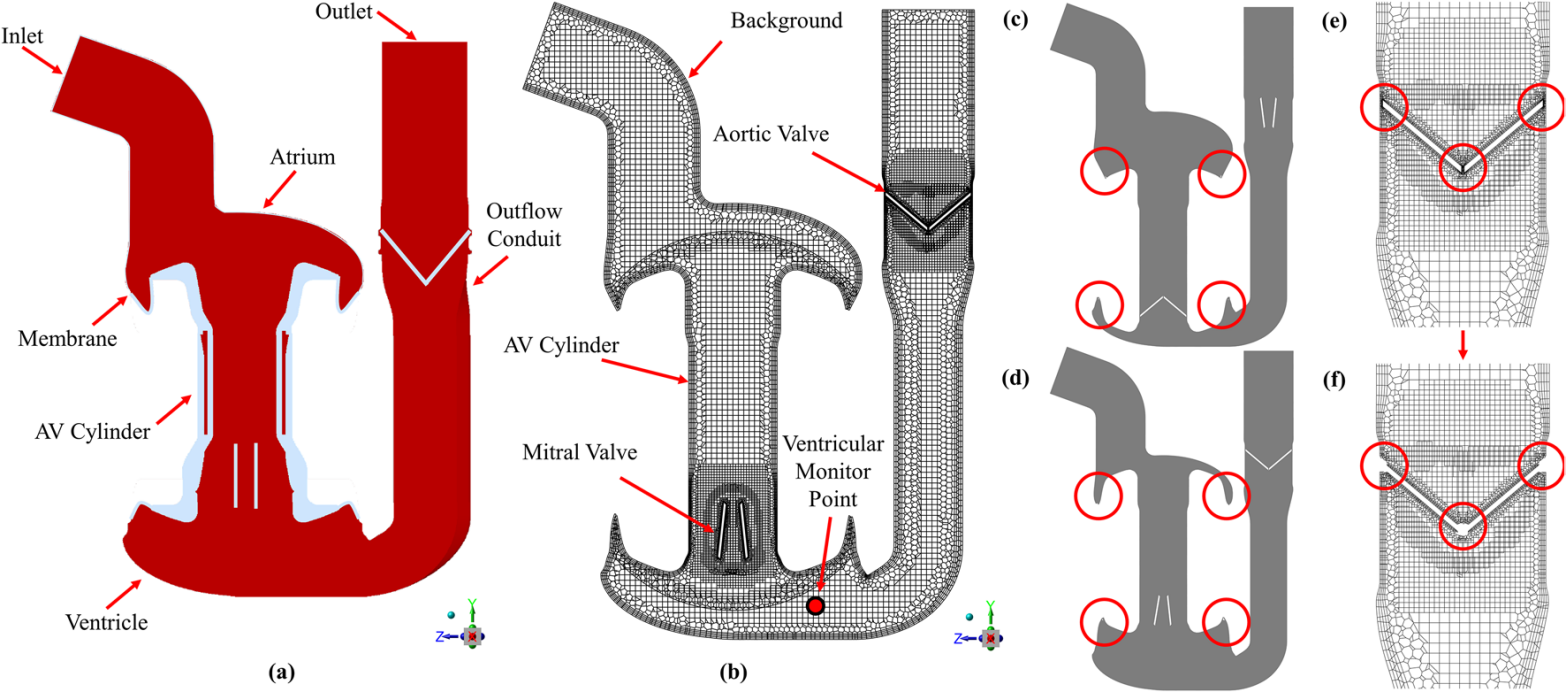
Computational domain and mesh of the Realheart TAH CFD model, showing (**a**) schematic of the TAH, with locations of the inlet and outlet, outflow conduit, atrial and ventricle regions alongside connective membrane linking the translating AV cylinder to these regions. (**b**) The internal mesh and the locations of the six overset component zones: background, AV cylinder, two mitral leaflets and two aortic leaflets. (**c**) Shape of the overlap between AV cylinder and atria and ventricle (circled in red) at end systole and (**d**) end diastole. (**e**) Locations of the small peripheral gaps (circled in red) that become excluded due to the gap model at (**f**) initialisation.

The computational model was developed for the Realheart V11c, a legacy prototype device that was used in acute bovine trials^40^, and has extensive in vitro data available for validation of the computational model. Newer versions of the Realheart TAH, both at prototype and concept stages, function similarly but have geometric variations, therefore it will be straightforward to extend the modelling to these after validation. The full device is comprised of two pumps, like the native heart. Each pump (Fig. 1a) has atrial and ventricular chambers, an outflow conduit that contains the aortic valve, and a cylindrical piston (AV cylinder) which functions as the AV plane, and houses the mitral valve. The AV cylinder is connected to the atrium and ventricle via a deformable membrane. Blood enters through an atrial inlet, where it fills the atria. The AV cylinder translates sinusoidally to push blood into the ventricle, increasing ventricular pressure, which in turn moves blood through the aortic valve and through the conduit outlet. The valves are ON-X BMHVs, which use an actuated pivot mechanism to allow for an opening angle of up to ${90}^\circ$ which promotes laminar flow, resulting in improved haemodynamics, a reduction in haemolysis and thrombogenicity^41^.

The model was created using the same method developed previously^39^, using an overset approach to combining the different moving parts. In the overset meshing approach, a static Eulerian background zone is used, with overlapping component zones that contain the moving solid parts. The interface between moving solids and the fluid is captured explicitly within the component zone, allowing for consistent and refined meshes at these locations. No mesh deformation occurs when the part moves, instead the entire component zone moves, and data is transferred between the overlapping and static meshes, similar to a Lagrangian FSI approach. Using Ansys Design Modeller V2021 R2 (Ansys Inc., Canonsburg, PA, USA), a total of six overset zones were created (Fig. 1b)—a background zone which contained the atrium, ventricle and outflow conduit, an AV cylinder zone and two zones per heart valve for each valve leaflet. The deformation of the membranes was not explicitly modelled. Instead, the shape of the deformation was approximated using the shape of the overlap of the walls of the AV cylinder zone and background zone (Fig. 1c,d). The pumping method of the device, with all the moving components, can be seen in the supplementary videos S1 through S3.

### Meshing

Each overset zone was meshed separately using Ansys Fluent Meshing V2021 R2 (Ansys Inc.) using a hybrid polyhedral and hexahedral mesh. Inflation layers were used on all walls to capture the fluid boundary layer. Local sizing was used on the surface of the valve leaflets and the faces directly surrounding the leaflets. Additionally, bodies of influence were used around the valves to obtain a finer mesh in this area.

A mesh convergence study was undertaken, and three meshes were generated representing a coarse, medium and fine mesh with total element counts of 280 k, 800 k and 4.5 m. Once the model was initialised, the total number of solve cells were 165 k, 480 k and 2.4 m.

### Hybrid cardiovascular simulator

A hybrid cardiovascular simulator, comprised of a lumped parameter computational domain that modelled the cardiovascular system and a physical hydraulic component featuring flow chambers, was adapted and connected to the Realheart TAH to form a loop, and the haemodynamic response of the device under physiologically realistic conditions was measured^22^. A pressure sensor (PPG Honeywell, Columbus, OH, USA) measured the left aortic pressure just downstream of the aortic valve, and a Transonic flow meter (ME24 PXN, T402 Transonic Systems Inc, Ithaca, NY, USA) measured the left outflow flow rates. Data was acquired at 1000 Hz through a National Instrument DAQ board and captured in LabVIEW 2019 (National Instrument, Austin, USA).

### Boundary conditions

A constant pressure boundary condition was imposed at the inlet. The value approximated pulmonary venous pressure and was obtained by using the time-averaged left atrial inlet pressure from the hybrid cardiovascular simulator. A two-element Windkessel model was applied at the outlet, approximating downstream vasculature, or in this case, the downstream loop of the hybrid cardiovascular simulator, and generated a physiological variation in outlet pressure^42^.

In the hybrid cardiovascular simulator, impedance values were set to a compliance $C = 0.6$ ml/mmHg and a resistance $R = 16.7$ Wood Units^22^. For the FSI simulation, the impedance parameters were the same as the experimental values, with the exception that the compliance was increased by an additional 0.2 ml/mmHg to account for parasitic compliances within the mechanical components of the hybrid cardiovascular simulator. The Windkessel pressure was initialised as the time-averaged aortic pressure observed in the hybrid simulator. No-slip conditions were placed on all walls of the model, and overset boundaries were used on the outer faces of the overset zones to enable the creation of the single continuous mesh.

### AV plane and valve motion

The five moving overset zones (translating AV cylinder, translating and rotating mitral valve leaflets and rotating aortic valve leaflets) were assigned user-defined functions that established the motion characteristics of each zone. The translation of the AV cylinder and mitral valve was described by a piece-wise sinusoidal function that produced different motion for downward (systolic) and upward (diastolic) translation. The ratio of the duration of systole to diastole was 40%:60%, equating to a shorter time in systole than diastole. The piece-wise velocities of the AV cylinder and mitral valve are outlined in Eq. (1) for the systolic and diastolic phases of the first cycle,

1 $$\begin{aligned} V_{AV} = {\left\{ \begin{array}{ll} \omega _{dias} A \text {cos}\left( \omega _{dias} \left( t - \frac{T_{dias}}{2} - T_{sys} \right) \right) &{} 0 \ge t< \frac{T_{dias}}{2} \\ -\omega _{sys} A \text {sin}\left( \omega _{sys} \left( t - \frac{T_{dias}}{2} \right) \right) &{} \frac{T_{dias}}{2} \ge t< \frac{T_{dias}}{2} + T_{sys} \\ \omega _{dias} A \text {sin}\left( \omega _{dias} \left( t - \frac{3T_{dias}}{2} - T_{sys} \right) \right) &{} \frac{T_{dias}}{2} + T_{sys} \ge t < T_{dias} + T_{sys} \\ \end{array}\right. } \end{aligned}$$

where $T_{dias}$ and $T_{sys}$ was the duration of the diastolic and systolic phases, and $\omega$ varied for systole and diastole ($\omega _{sys} = \pi /T_{sys}$ and $\omega _{dias} = \pi /T_{dias}$). This equation allowed for the variation of the pumping amplitude *A*, mm, the pumping frequency or heart rate *f*, bpm, and the systolic–diastolic ratio. The value of *A* was determined from the diagnostic data output from the pump during the in vitro study, where *A* was derived from the AV plane displacement over time. To match the experimental data from the hybrid simulator, *A* and *f* were varied to create 16 different operating conditions at stroke lengths of 19–21–23–25 mm and heart rates of 60–80–100–120 bpm.

The rotation characteristics of the valve leaflets were defined in the set of user-defined functions, which assigned the mass, moment of inertia and centre of rotation of each leaflet. Based on the dimensions of the leaflets and a material of pyrolytic carbon, each leaflet was approximately 0.35 g, giving rise to a moment of inertia of $2\times 10^{-8}$ kg $\hbox {m}^2$, and had one degree of rotation around the *x* axis. As per the previously developed valve motion method, the pivot mechanism of the bileaflet valve was neglected and a maximum opening angle of ${84}^\circ$ was used^39^. The leaflets were constrained between ${40}^\circ$ and ${84}^\circ$ when fully closed and fully open respectively. The centre of rotation of the aortic valve was fixed in space, whilst the centre of rotation of the mitral valve moved with the AV displacement over time.

### Numerical procedure

Ansys Fluent V2021 R2 (Ansys Inc.) was used to solve the equations for the fluid flow, and the in-built six degrees of freedom solver was used for the rigid body motion of the valve leaflets.

In the model that we developed previously^39^, the fluid flow and rigid body motion were coupled using a blend of weak explicit and strong implicit phases to improve computational stability and efficiency. For this study, the strong coupling was modified to be enabled when the angle of any valve leaflet was less than ${84}^\circ$ and the leaflet angular velocity was more than 0 rad/s, with a motion relaxation factor of 0.4. This change was made to improve solution stability, as instabilities were encountered using the same approach as previously taken. Weak coupling was therefore enabled only when the leaflets were stationary, defined as the angular velocity of all valve leaflets being 0 rad/s.

The peak Reynolds number was calculated to be 9100 in the outlet conduit of the device using the peak flow rate at a heart rate of 120 bpm and a stroke length of 25 mm. This placed it in the turbulent regime, and as such the unsteady Reynolds Averaged Navier–Stokes (RANS) equations were solved with the SST $k-\omega$ model for turbulence closure, an approach taken by others when simulating artificial hearts^32,37^.

In reality, there will be a non-perfect contact between the valve leaflet and valve housing when the leaflet is in the fully closed position, creating a thin peripheral gap. Such an effect was not modelled in this case to avoid very high mesh density in this region and improve computational efficiency. Instead, the *gap model* function within Fluent was used, where a threshold gap distance was defined between the leaflets and the housing, as well as the middle gap between the leaflets, whereby below this distance, the elements would be blocked off, and a perfect seal would be assumed (shown in Fig. 1e,f).

The coupled solver was used for the RANS equations, using a least-squares gradient scheme, and second order pressure and momentum schemes. Solution relaxation with a value of 0.75 was used for pressure and momentum schemes, as well as higher order terms. This improved convergence and stability of the solution. Blood was approximated as Newtonian, with a density $\rho =$ 1060 kg/$\hbox {m}^3$ and fixed viscosity of $\mu =$ 3.5$\times 10^{-3}$ N/m $\hbox {s}^2$^43^.

A passive Eulerian scalar transport model was included, where the transient advection equation was solved for the washout of blood throughout the device. At the inlet, a value of 1 was assigned, which equated to new blood, and the scalar field was initialised with a value of 0.

Each condition was simulated for five cycles on a Microsoft Azure cloud HPC system operated by the University of Bath^44^, using 32 Intel Xeon Platinum 8168 cores, and took an average 1440 core hours to complete. Contour plots were created in Ansys Fluent. Data was plotted using MATLAB (Release 2020b, The MathWorks Inc., Natick, Massachusetts). Cyclic convergence was assessed by calculating the cycle-to-cycle root mean square error (RMSE) of the outlet volume flow rate and aortic pressure, where a decrease in RMSE with an increase in the number of cycles indicated cyclic convergence, resulting in five cycles being sufficient for convergence. Washout results were extracted at the end of four cycles to compare to other studies^34,38^, whilst shear stress data was obtained during the fifth cycle.

## Results

### Mesh study

**Figure 2:**
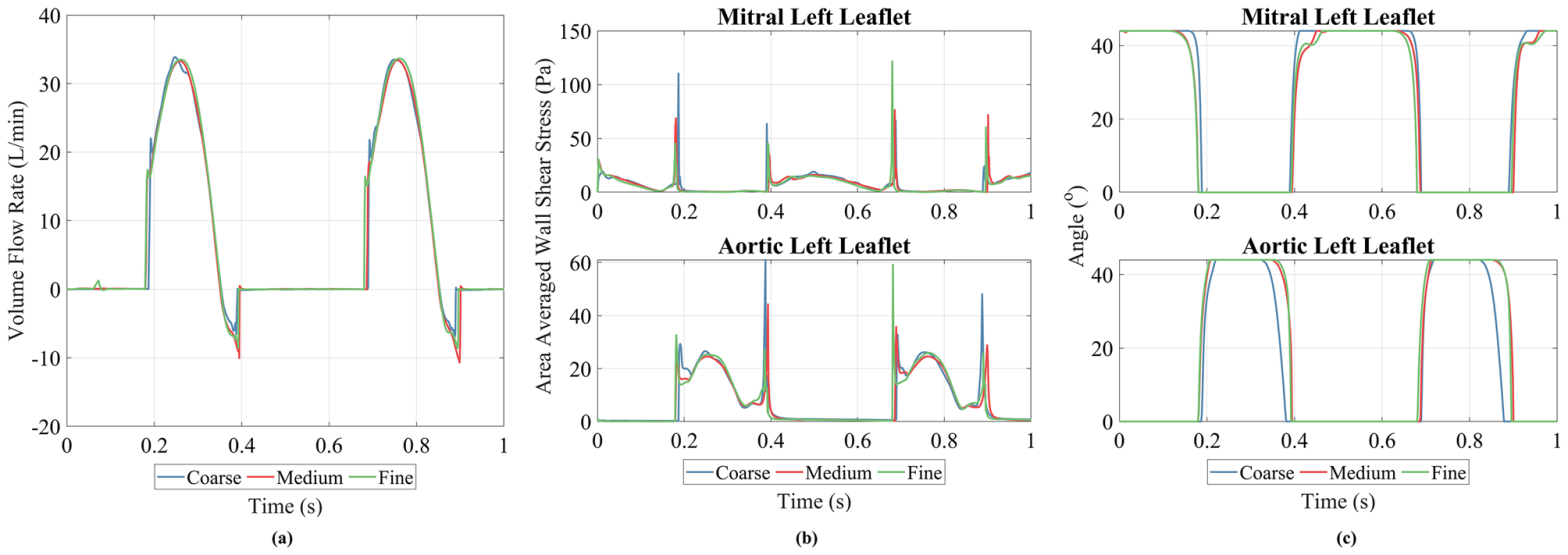
Mesh study results for the coarse, medium and fine meshes, showing (**a**) transient outlet volume flow rate, (**b**) transient leaflet angle for the left mitral and left aortic valve leaflet and (**c**) area-averaged transient wall shear stress for the left mitral and left aortic valve leaflets.

The three meshes were compared using the variation in outlet volume flow rate (Fig. 2a), valve leaflet angle (Fig. 2b) and area-averaged wall shear stress on the valve leaflets (Fig. 2c) over the course of two cycles at a heart rate of 120 bpm and a stroke length of 25 mm. In all cases, the medium and fine meshes were very similar, whilst the coarse mesh displayed some small differences, most notably in valve leaflet angle. The RMSE between the medium and fine mesh was 1.74 L/min (5.2% of the peak value) for the volume flow rate, ${3.6}^\circ$ and ${3.1}^\circ$ (8.1% and 7.0% of the peak value) for the valve leaflet angle, and 7.9 Pa and 5.1 Pa (6.5% and 8.6% of the peak values) for the area-averaged wall shear stress. In all cases the coarse mesh returned greater RMSE values. The total wall clock time for each mesh was 9 h, 18 h, and 92 h. Considering simulation accuracy, as well as stability and simulation time, the medium mesh was considered sufficient to use for the study.

### Flow field

**Figure 3:**
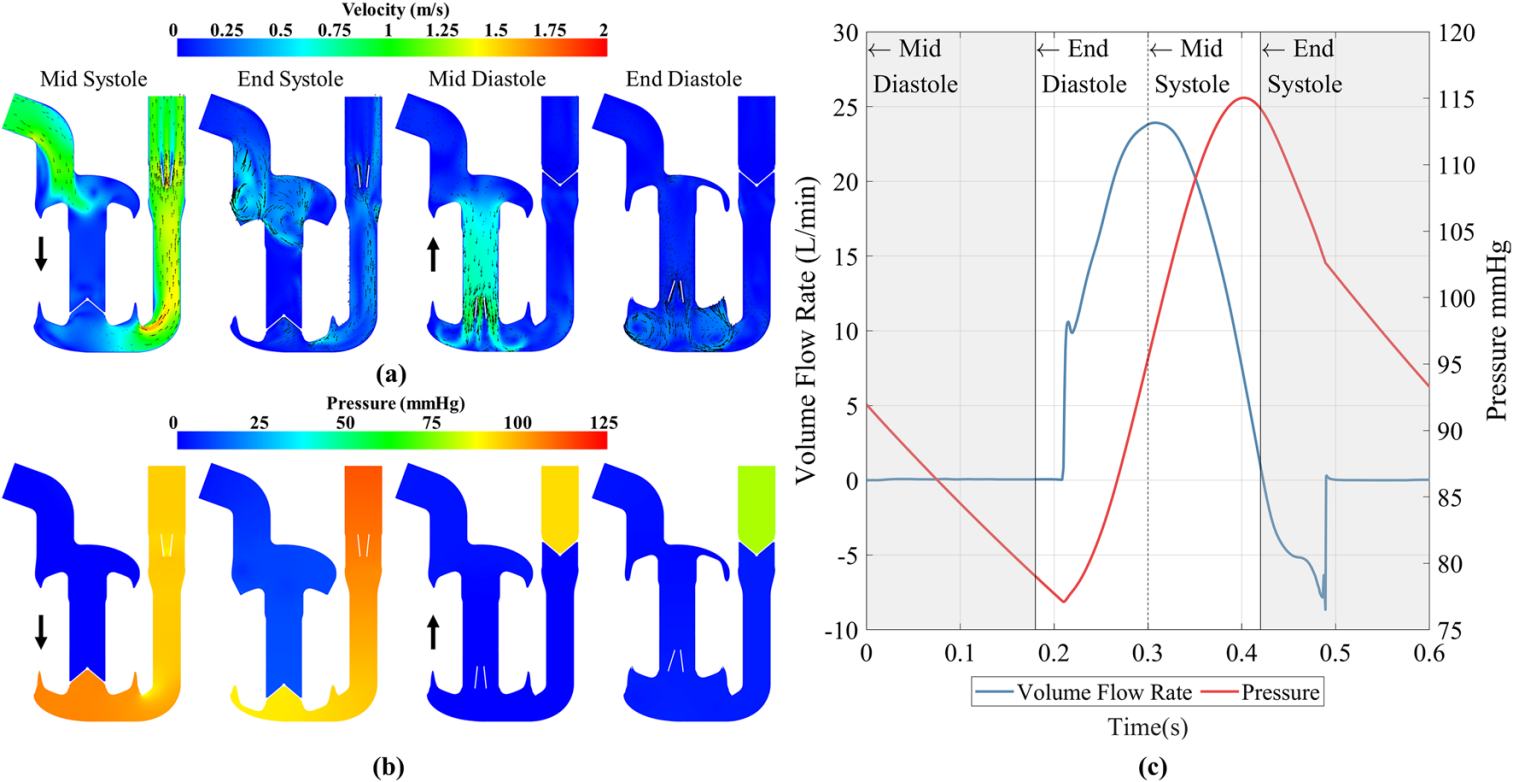
(**a**) Velocity flow field and (**b**) pressure contour plots at mid systole, end systole, mid diastole and end diastole at an operating point of 100 bpm 21 mm, equating to 5 L/min. Black arrows at mid systole and mid diastole denote direction of the AV plane. (**c**) Transient outlet volume flow rate and outlet pressure. Grey regions of plot denote diastole whilst white regions are systole.

Cyclic convergence was assessed using RMSE: at an operating point of 100 bpm and 21 mm, equating to 5 L/min, RMSE as a percentage of the peak value for the outlet volume flow rate dropped from 9.4% between the first and second cycle, to 5.6% between the fourth and fifth cycle, whilst RMSE for aortic pressure dropped from 0.8 to 0.2%. The fifth cycle was then considered to be cyclically converged. An example of the temporal variation in pressure and velocity contour plots, flow rate and aortic pressure is shown in Fig. 3, at an operating point of heart rate at 100 bpm and a stroke length of 21 mm. Animations of the velocity, pressure, washout and mesh motion can be found in Supplementary videos S1 through S3 for low, medium and high cardiac outputs. The cycle started at mid diastole, where the AV cylinder was translating upwards. Two counter rotating vortices were observed in the ventricle as the blood moved down past the mitral valve. The mitral valve closed at the beginning of systole when the AV cylinder began to move downwards, which created a positive pressure gradient across the aortic valve, causing it to open. During systole, new blood was drawn in through the inlet which created a vortex in the centre of the atrium. Blood was pushed from the ventricle through to the outlet, where it accelerated past the ventricle-outflow junction, up the outflow conduit and past the aortic valve, creating a prominent three-jet structure as it passed the valve. Peak outlet flow rate was observed at mid systole, where the AV cylinder was translating down at maximum velocity, and peak outlet pressure occurred just before end systole. The aortic valve closed at the beginning of diastole due to backflow drawn through the outlet. Once it was closed, a negative pressure gradient across the mitral valve was created which caused it to open, and the cycle restarted.

### Hybrid cardiovascular simulator comparisons

**Figure 4:**
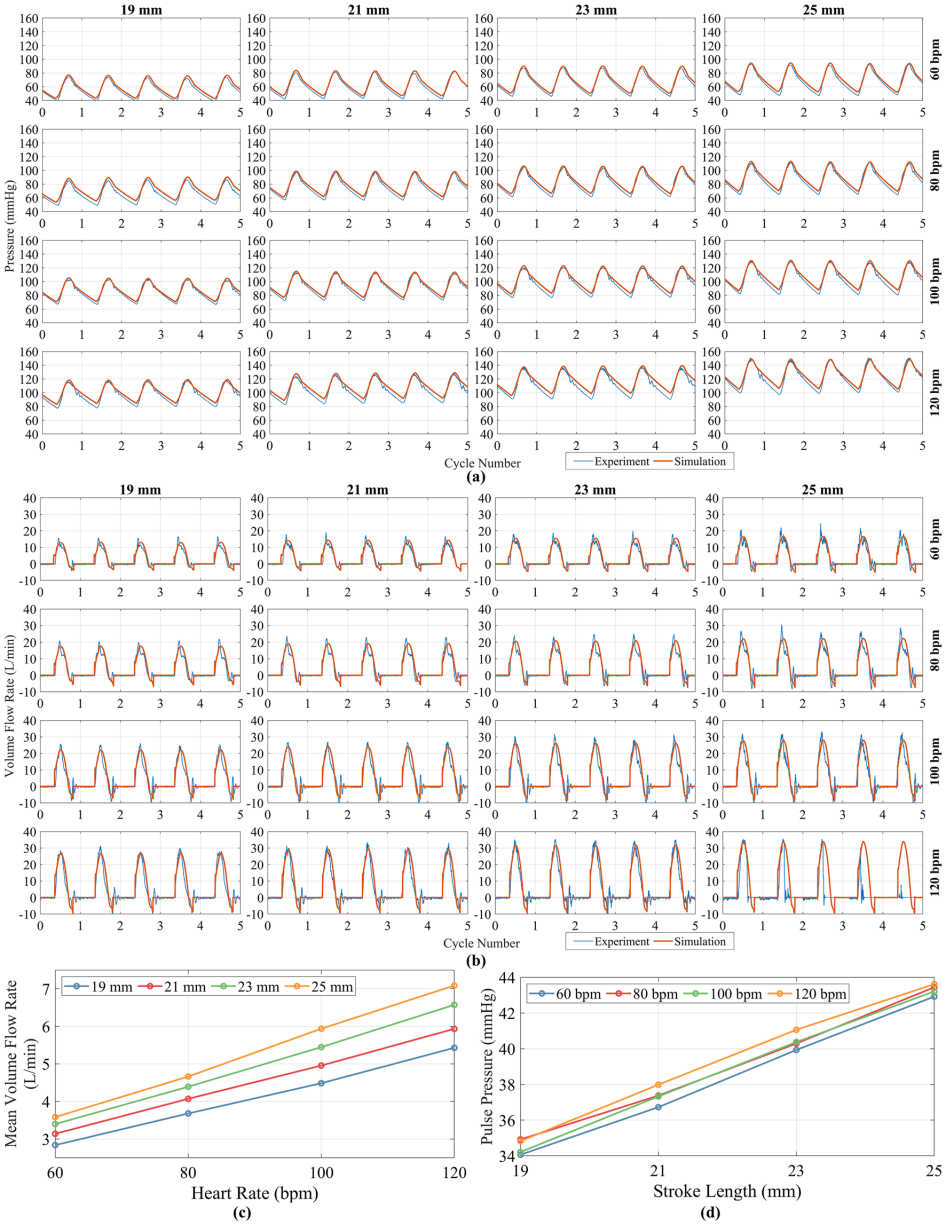
Comparison between simulated and experimental data from hybrid cardiovascular simulator of (**a**) outlet pressure and aortic pressure and (**b**) outlet volume flow rate and outflow volume flow rate showing good qualitative agreement between the sets of data. (**c**) Mean outlet volume flow rate against heart rate for a change in stroke length. (**d**) Outlet pulse pressure against stroke length with a change in heart rate.

The transient volume flow rate, $Q_{\text {out}}$ (L/min) and pressure, $P_{\text {out}}$ (mmHg) at the outlet was compared to the in vitro data obtained from the hybrid rig for the 16 different operating points that were considered (Fig. 4a,b). Qualitatively, there was good agreement between the simulated and experimental pressure and flow rate. Small differences in flow rate were observed at the end of each pulse when the experimental results oscillated more than simulated results, which may be attributed to model simplifications compared to the entire hybrid cardiovascular simulator; specifically waves are reflected from the rigid downstream components. Quantitatively, the maximum RMSE for $P_{\text {out}}$ as a percentage of peak $P_{\text {out}}$ was 5%, occurring at 80 bpm 19 mm. The maximum RMSE for $Q_{\text {out}}$ as a percentage of peak $Q_{\text {out}}$ was 15%, occurring at 100 bpm 19 mm. The relationships between mean $Q_{\text {out}}$ and pulse pressure with stroke length and heart rate are characterised in Fig. 4c,d. Mean $Q_{\text {out}}$ increased linearly with an increase in heart rate and stroke length, with a maximum value of 7.1 L/min at 120 bpm 25 mm, and a minimum value of 2.8 L/min at 60 bpm 19 mm. The pulse pressure increased linearly with stroke length, but remained mostly constant with a variation in heart rate. The maximum pulse pressure was 43.6 mmHg, also occurring at 120 bpm 25 mm, whilst the minimum was 34 mmHg, also occurring at 60 bpm 19 mm.

### Valve kinematics

**Figure 5:**
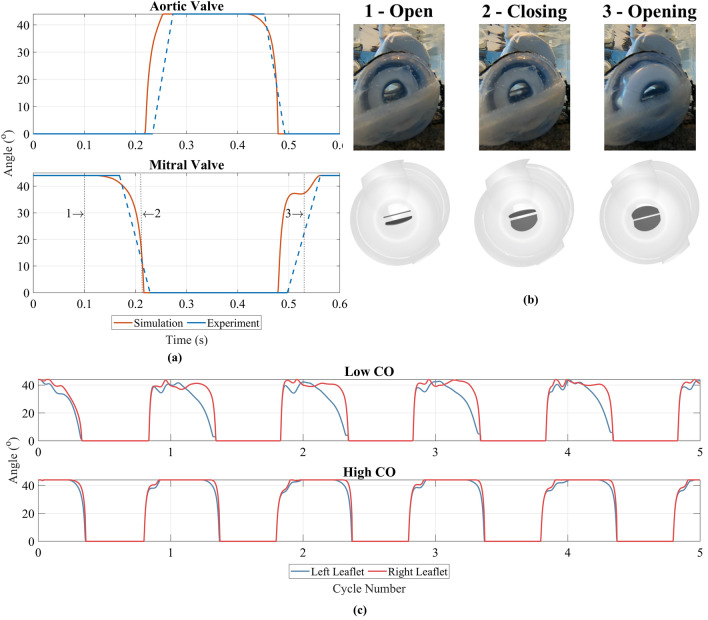
(**a**) Transient comparison of the aortic and mitral valve angles between simulated and in vitro data. The simulated left valve angle was used in both cases. Solid blue lines represent fully open and fully closed. Dashed blue lines represent the transition between the two states and not actual valve motion characteristics. (**b**) Image comparison between the in vitro video capture and simulated mitral valve position. Numbered points correspond to points on (**a**), where point 1 is mitral valve fully open, point 2 is during closing of the mitral valve and point 3 is during opening of mitral valve. Mitral valve leaflets are shown in dark grey, whilst light grey is rest of pump housing. (**c**) Comparison of the left and right mitral valve leaflets for the lowest CO (60 bpm 19 mm) equating to 3 L/min, and the highest CO (120 bpm 25 mm) equating to 7 L/min, where ${0}^\circ$ is fully closed and ${44}^\circ$ is fully open.

Data from videos of the in vitro valve motion recorded at a frequency of 200 Hz^38^, with the operating condition 100 bpm 25 mm, were compared with the valve motion from the simulations (Fig. 5a). Both valves were seen to start to close and finish closing at similar times. Duration of fully open and fully closed stages for both valves are also similar, with only a 0.01 s difference (equivalent to 2 frames of video data) in both cases. Three time points were selected and the mitral valve was visually compared between the two sets of data in Fig. 5b. Similar opening and closing behaviour was observed, notably the ‘plateauing’ of the mitral leaflet angle upon opening, which occurred in both sets of data. It was not possible to measure exact angles from the videos. The start time and end time of the opening phase were very close in the in vitro and simulated data, however the images reveal some discrepancy between the angles of the mitral valve leaflets in the video data and simulated data.

There was some variation in the behaviour of the simulated kinematics of the mitral valve leaflets between operating points when the valve was in the open phase. Figure 5c shows the variation in angle for the left and right mitral leaflet for the lowest and highest cardiac outputs (CO). Oscillations in both the left and right leaflet angles were observed at the low CO, and the left leaflet started to close at a slightly earlier time. This asymmetric behaviour of the valve leaflets was not as prominent at a high CO, where instead the two leaflets began to close at similar times, and did not display the same oscillating behaviour during the fully open phase. This behaviour was not observed for the aortic valve. Instead the opening and closing of both aortic valve leaflets occurred at the same time, and no oscillation was present during the fully open phase.

### Washout

**Figure 6:**
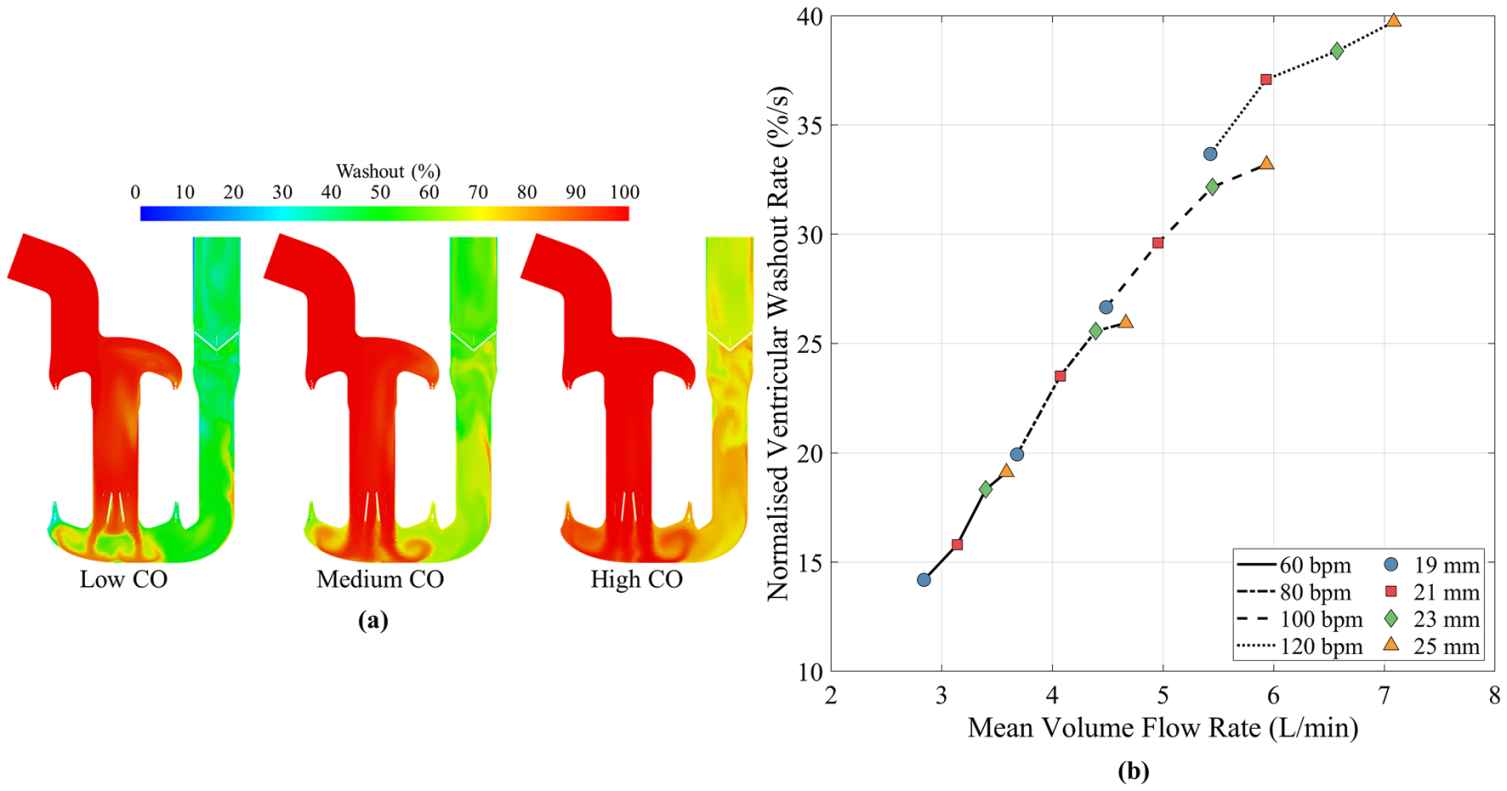
(**a**) Washout contour plots for low (60 bpm 19 mm), medium (100 bpm 21 mm) and high (120 bpm 25 mm) CO, equating to 3, 5 and 7 L/min. (**b**) Rate of ventricular washout against mean volume flow rate for the 16 simulated operating points.

The contours of washout after four cycles for low, medium and high CO operating points are shown in Fig. 6a. This showed an increase in washout with an increase in CO. The majority of the atrial region was fully washed out in the three conditions, with the right-hand side of the atrium and the top of the AV cylinder below 100% at the low and medium CO. The ventricle and outlet conduit regions displayed the greatest spatial variation in washout between the three conditions, with washout in these areas increasing as CO increases. Areas of lowest washout were seen at the boundary between the AV cylinder and the ventricle region, however new blood was continuously entering this area during diastole. The washout scalar was calculated at the ventricular monitor point defined in Fig. 1b. The ventricular washout at the end of the four cycles was 64%, 80% and 89% for the three COs respectively.

To compare the washout performance of each operating point, the ventricular washout value at the end of four cycles was normalised by the total time, arriving at a ventricular washout rate, shown in Fig. 6b. A mostly linear trend in washout rate with respect to mean volume flow rate was observed, especially at lower flow rates. A maximum rate of approximately 40%/s occurred at 120 bpm 25 mm. The washout rate levelled off at high stroke lengths, with the magnitude of the levelling off increasing as heart rate increased.

### Shear stress

**Figure 7:**
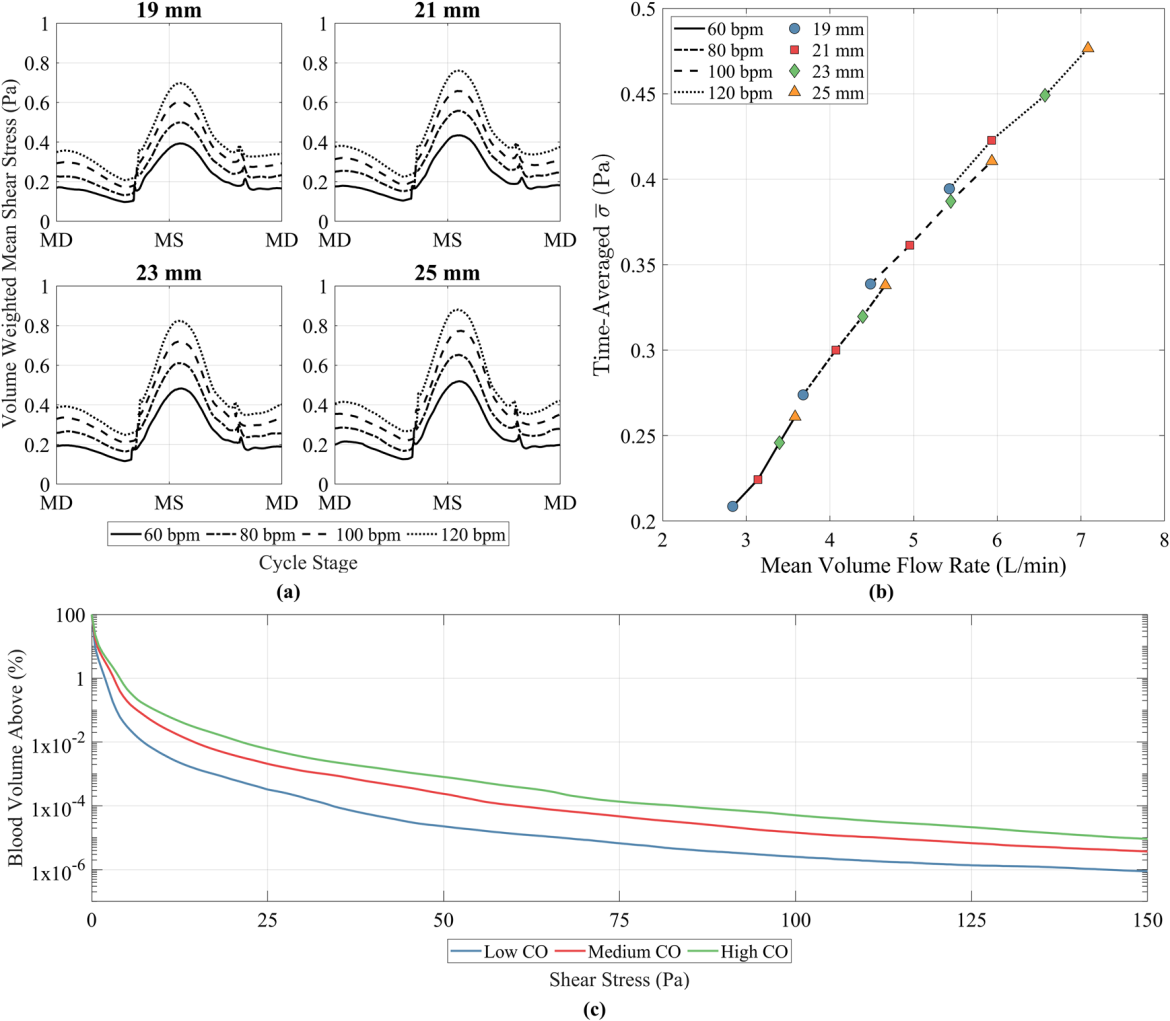
(**a**) Volume-weighted mean scalar shear stress (${\overline{\sigma }}$) against time for the different conditions simulated. MD refers to mid diastole whilst MS refers to mid systole. (**b**) Time-averaged ${\overline{\sigma }}$ against mean volume flow rate for each of the conditions simulated. (**c**) Average percentage volume of blood exposed to a given shear stress over the course of one cycle. Low CO was 60 bpm 19 mm (3 L/min), medium CO was 100 bpm 21 mm (5 L/min) and high CO was 120 bpm 25 mm (7 L/min).

The temporal variation in shear stresses was investigated using the volume-weighted mean scalar shear stress, ${\overline{\sigma }}$ (Pa), which was calculated using Eq. (2) for each operating point at every time step,

2 $$\begin{aligned} {\overline{\sigma }} = \frac{1}{V} \int \sigma d V = \frac{1}{V} \sum ^{n}_{i=1} \sigma |V_i| \end{aligned}$$

where *V* was the total pump volume, $\sigma$ was the scalar shear stress, *n* was the number of fluid elements and $V_i$ was the volume of a given fluid element. The variation in ${\overline{\sigma }}$ over time is shown in Fig. 7a.

${\overline{\sigma }}$ was greater during systole than diastole. The peak value occurred just after mid systole, where the magnitude of the peak increased with an increase in stroke length and heart rate. The minimum ${\overline{\sigma }}$ occurred at end diastole, just before the mitral valve closed and the aortic valve opened. The opening and closing of the valves created a small spike in ${\overline{\sigma }}$. The peak ${\overline{\sigma }}$ during diastole occurred just after mid diastole, and again this value increased with an increase in stroke length and heart rate. The relationship between average volume flow rate and the time-averaged ${\overline{\sigma }}$ is displayed in Fig. 7b. A linear trend is seen, where time-average ${\overline{\sigma }}$ increases with mean volume flow rate. For operating points which produced an equivalent average flow rate, a lower time-average ${\overline{\sigma }}$ was observed by increasing the stroke length and decreasing the heart rate.

The spatial variation in shear stresses was investigated using a cumulative exposure approach, where the total blood volume above a given shear stress threshold was calculated for the three CO conditions at each time step and averaged over time to arrive at a mean blood volume for each shear stress threshold, shown in Fig. 7c. Mean volumes exposed to elevated stress levels increased as CO increased, but the bulk of the stresses experienced were low, with 99% of the mean volume exposed to below a shear stress of 2, 3 and 4 Pa for the low, medium and high CO respectively. Two threshold values of 17.5 Pa and 150 Pa were used to investigate exposure to an elevated shear stress in both space and time, and correlated to damage of the von Willebrand Factor and red blood cells respectively^35,43^. The mean percentage volume exposed to above 17.5 Pa was 0.001%, 0.006% and 0.018% for the three conditions. The percentage cycle during which these conditions were exceeded was 97% at low CO, 93% at medium CO and 100% for high CO. The mean percentage volume exposed to above 150 Pa was 9.0 $\times 10^{-7}$%, 3.7 $\times 10^{-6}$% and 9.2 $\times 10^{-6}$% for the three conditions, with a maximum value of 1 $\times 10^{-4}$%, 2.3 $\times 10^{-4}$% and 4.5 $\times 10^{-4}$% respectively. The percentage cycle time during which 150 Pa was exceeded was 2.7%, 10.0% and 16.9% for the three conditions respectively.

**Figure 8:**
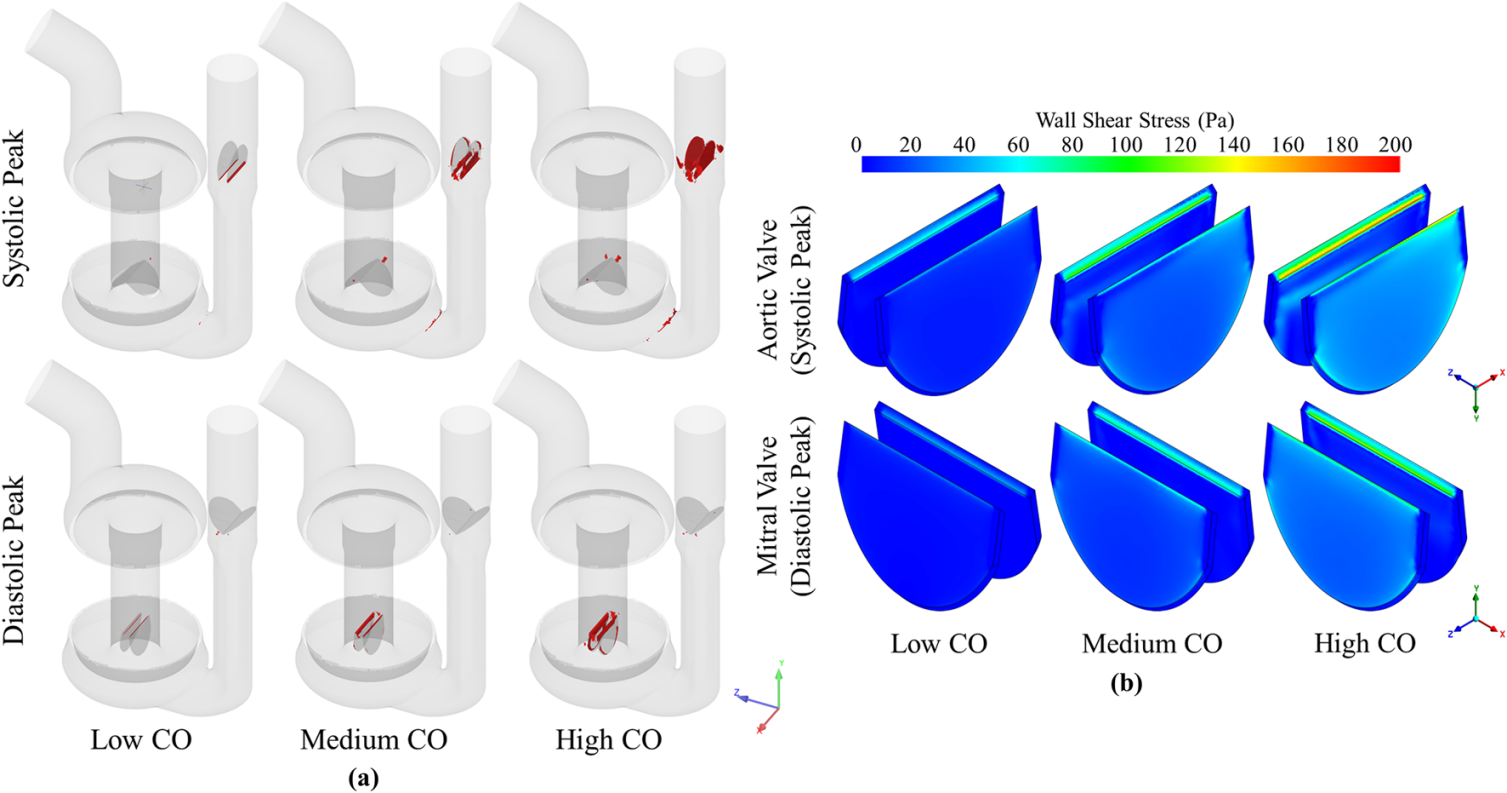
(**a**) Volume of fluid that exceeds the threshold scalar shear stress value of 17.5 Pa at systolic and diastolic peak scalar shear stress time points and (**b**) wall shear stress distribution of aortic valve at systolic peak scalar shear stress, and mitral valve at diastolic peak scalar shear stress time points for low (60 bpm 19 mm), medium (100 bpm 21 mm) and high (120 bpm 25 mm) CO, equating to 3, 5 and 7 L/min.

The time points corresponding to the peak ${\overline{\sigma }}$ during systole and diastole were chosen to investigate the spatial distribution of the scalar shear stresses. The locations of the blood that exceeded the threshold shear stress of 17.5 Pa at the two time points are shown in Fig. 8a for the three CO cases. As stated previously, the volume of fluid exposed to above 17.5 Pa increased as the CO increased. At the systolic ${\overline{\sigma }}$ peak, the bulk of the elevated shear stress was located around the aortic valve as blood accelerated around the valve leaflets. In addition, at the junction between the ventricle and the outlet conduit, there existed a region of elevated shear stress where fluid accelerated through the constriction between these two regions. During the diastolic ${\overline{\sigma }}$ peak, the elevated shear stress was located around the mitral valve, as the AV cylinder translated upwards and blood moved downwards past the mitral valve. Figure 8b shows the surface distribution of the wall shear stress of the aortic valve at the corresponding time points. The leading edge of the valve leaflet in both cases was the location of the highest wall shear stresses, which were larger in the case of the aortic valve.

## Discussion

The Realheart TAH can be operated in a variety of ways to achieve many possible pulsatile pumping characteristics. The effect of these pumping characteristics on the blood and the rest of the body may not be intuitively obvious, and as such, a computational model that can provide an in-depth assessment of some of these effects is beneficial. The aim of this study was to produce such a CFD model, and measure how a variation in stroke length and heart rate affected the pumping waveform, valve kinematics, washout and shear stresses.

Whilst the operation of the TAH can be prescribed by the user, the behaviour of certain core device components are reactive to a change in these input parameters. The pair of BMHVs contained within the device govern the forward motion of blood, and open and close due to the fluid forces acting on them. BMHVs are traditionally used in aortic or mitral valve replacement, and as such exist in elastic chambers that comply with the surrounding flow. In this application, both sets of valves exist in a more constrained settings, thus giving rise to the potential for differing flow characteristics. In this study, an FSI valve motion strategy that had been developed previously^39^ was implemented into the model of the full device. This strategy considered the fluid forces acting on the valve leaflets, causing them to open and close during the cycle. This work is an improvement upon the previous approach taken by Kelly et al.^38^, who prescribed the motion of the valve leaflets based on video analysis. This study has shown that the valve leaflets, notably those of the mitral valve, behave differently under different operating conditions. The oscillatory behaviour at lower COs resulted in cycle-to-cycle variation in leaflet motion for the five cycles simulated. This behaviour will impact processes such as washout as blood will transit between the atria and ventricle in slightly different ways from cycle to cycle. This would not be the case at high COs, as the mitral valve leaflet behaviour was more consistent between cycles. The ability to capture this valve leaflet behaviour will ultimately lead to more accurate results of secondary analyses such as washout and blood damage predictions. The valve opening behaviour was similar to that seen by Mirkhani et al for the ON-X valve under physiological conditions in an ascending aorta^45^. At an operating condition of 6 L/min, the valve opening time of Mirkhani et al was approximately 80 ms, compared to approximately 60 ms in this study. Additionally, both studies displayed similar flow field characteristics, with a prominent but simple three-jet structure past the fully open valve leaflets, also observed by Akutsu and Matsumoto^46^.

Whilst valve durability was not the focus of this work, ON-X valves have been tested, approved, and used not only in the aortic position but in the mitral position as a heart valve replacement. Even in these scenarios where the expected therapy is much longer than in TAH applications, excellent results have been shown with regards to valve durability^47^. As stated previously, the Realheart pumping principle resulted in similar flow profiles around the valves and therefore durability of the valves is not a major concern. Nevertheless, there is the option to adjust the motor control to reduce transient pressure peaks during valve closure, and the durability will be verified in the final system tests.

The simulated results of $Q_{\text {out}}$ and $P_{\text {out}}$ were compared to those of the TAH in the hybrid cardiovascular simulator. The excellent agreement between the sets of results highlights the accuracy and adaptability of the model to a change in operating condition, indicating that in future studies the model could be simulated across additional operating points that have not been considered here. Not only this, but different generations of the device can be created using the same modelling approach, including those that have not been prototyped, paving the way for accurate performance predictions and comparisons between generations. Additionally, whilst the two-element Windkessel model approximated systemic circulation in this study, this could be made patient-specific in future studies, allowing for a more personalised physiological pressure response from the simulation.

The experimental $Q_{\text {out}}$ value displayed oscillations, particularly after aortic valve closure, whereas the simulated $Q_{\text {out}}$ had a much smoother numerical response. This could be attributed to the downstream fluid chambers of the hybrid cardiovascular simulator not being explicitly modelled in the CFD, where reflected waves could create small variations in flow rate over time. Another reason may be the omission of the flexible membranes within the device, which would also contribute to small transient variations in flow rate. However, the general shape of the pumping waveform agrees well, with peak values not varying considerably between the simulated and experimental data.

The non-physiological flow conditions that arise within mechanical circulatory support devices such as VADs or TAHs will lead to some level of blood trauma, be it damage to the blood components or thrombogenic events^48^. Thrombus formation can occur when activated platelets are deposited in regions of low shear, where the blood is stagnant. Washout simulations can offer insight into how blood transits the device, highlighting any potential areas where blood may stagnate and how this varies with a change in operating conditions.

Washout was simulated using an Eulerian scalar transport model, similar to other approaches to simulating washout in VADs^49,50^ and TAHs^38^. Other approaches have used a volume of fluid method to treat the old and new blood phases separately^34,37^. The Eulerian approach was used here to maximise the flexibility of the model, so that any future numerical analyses could be undertaken on a single phase flow field.

In this study, the washout at the end of four cycles was normalised by total time to arrive at a washout rate, enabling the comparison of different heart rates. A higher level of washout rate is desirable, as it directly translates to a reduction in blood stagnation time, reducing the possibility of thrombus formation. Washout is a non-linear process however^49,50^, comprised of an initial linear increase at low values of washout and a non-linear increase in washout at higher values, where the rate of washout decreases. The washout rate calculated in this study would assume a linear relationship between washout and time, and may describe why the washout rate tapered off in Fig. 6a, where higher volume flow rates produce larger washout values that exist in the non-linear regime. Future work could investigate the time taken to reach 95% washout for each condition, however due to the pulsatile nature of the pump, this may take many cycles to achieve, especially at low mean volume flow rates. In addition, the influence of reducing residual volumes in the atria and ventricles will be of high interest.

An improvement in washout rate was seen with an increase in cardiac output. However for operating points that generated the same cardiac output (within 3% of one another), improved washout rate was achieved with increased heart rate but decreased stroke length, such as 100 bpm 23 mm and 120 bpm 19 mm.

Compared to the previous Realheart CFD study by Kelly et al.^38^, washout behaviour was similar, with the same ventricular washout percentage after 4 cycles (87% compared to 86% in this study) for the same operating point (80 bpm 25 mm). Improved washout was seen in this study at the overlap of the AV cylinder and the atrial and ventricular regions. This difference is likely due to the differences in the computational models, as the overset method used in this study has defined solid-fluid interfaces, whereas the immersed boundary method used previously does not. When comparing the results of this study to the CARMAT, the washout compares well, where the percentage washout was 93.8% and 85% in the left and right ventricle respectively^34^.

Damage to blood components occurs due to an exposure to elevated shear stresses over time^51^. Short of modelling the damage to blood explicitly using a damage function, investigating the distribution of shear stresses and the length of time for which a threshold stress has been breached is a good proxy for blood damage analysis^52^. The bulk of the shear stresses were of a low magnitude, so an investigation of a lower shear stress threshold could provide useful insight into other types of blood component damage. A value of 17.5 Pa was used, as this has been linked to the deterioration of the von Willebrand Factor^53^. Whilst exposure above 17.5 Pa was consistent throughout the cycle for the three conditions considered, the mean percentage blood volume that was exposed to above 17.5 Pa was low for all conditions, with a maximum value of 0.018% of the mean volume exposed at the highest CO (7 L/min). These values are lower than reported for the CARMAT, where for similar COs, the mean percentage volumes exceeded were 0.004%, 0.02% and 0.03% respectively^35^.

The second threshold value investigated in this study was 150 Pa, which has been associated with haemolysis of red blood cells^43,51,54^. This value was not breached for the majority of the cycle in all cases. When it was breached, the volumes of fluid that exceeded this threshold were very small, often equating to only a few cells at any given time step, meaning the likelihood of damage to red blood cells was low.

By investigating the relationship between time-averaged ${\overline{\sigma }}$ and operating point, optimisations to the operation of the device could be suggested. As with washout, operating points that produced the same mean volume flow rate were compared. This suggests that a small decrease in time-averaged ${\overline{\sigma }}$ can be achieved by increasing the stroke length and decreasing the heart rate. This points towards the same conclusion drawn by both Syncardia^32^ and ReinHeart^55^: an increase in heart rate due to downsizing the pump resulted in higher levels of shear stress and haemolysis. The method of reducing shear stresses observed in this study is at odds with the outcome found for increasing washout rate, suggesting that a trade-off would have to be made between shear stress levels and washout performance.

In this study, a gap model was used to numerically disregard the peripheral gaps around the outside of the valve leaflets, as well as between the leaflets. This created a perfect valve seal, and as a result, leakage flow was neglected. Leakage flow, especially through the hinge region of a BMHV has been shown to create regions of very high shear^56–58^. In future studies, particularly those that focus on the damage to blood components, the fidelity of the valves in the model can be improved, which should lead to more accurate blood damage predictions. Another model simplification was the omission of explicit modelling of membrane deformation. The addition of the deforming membrane would absorb pressure increases within the atrial and ventricle regions, potentially causing a small change to valve motion, pressure and volume flow rate characteristics, and altering the washout characteristics in the region near the AV cylinder.

To conclude, the FSI model of the Realheart TAH has been shown to be accurate and robust across a large range of clinically relevant operating conditions, with excellent agreement with in vitro experiments conducted on a hybrid cardiovascular simulator. The model is able to capture the washout of blood throughout the device, and shows agreement in behaviour with other similar studies. Additionally, investigations into the spatial and temporal distribution of scalar shear stresses highlight a low possibility for blood damage, due in part to very low volumes of blood exposed to elevated stresses. This FSI model can be applied to other positive-displacement artificial hearts that use mechanical valves to govern forward motion of the blood. Future work will quantify the level of haemolysis experienced within the device, as well as the potential for thrombosis.

## Supplementary Information

Supplementary Video 1.

Supplementary Video 2.

Supplementary Video 3.

## Data Availability

The datasets generated and/or analysed during the current study are not publicly available due commercial sensitivity but are available from the corresponding author on reasonable request.
